# Long-term outcomes after acute hypercapnic COPD exacerbation: First-ever episode of non-invasive ventilation

**DOI:** 10.1007/s00508-018-1364-6

**Published:** 2018-07-31

**Authors:** Andreas S. Fazekas, Mei Aboulghaith, Ruxandra C. Kriz, Matthias Urban, Marie-Kathrin Breyer, Robab Breyer-Kohansal, Otto-Chris Burghuber, Sylvia Hartl, Georg-Christian Funk

**Affiliations:** 1 Department of Respiratory Medicine and Critical Care, Otto Wagner Hospital, Baumgartner Höhe 1, 1140 Vienna, Austria; 2 Ludwig Boltzmann Institute for COPD and Respiratory Epidemiology, Vienna, Austria; 3 Medical University of Vienna, Vienna, Austria

**Keywords:** COPD, COPD exacerbation, Non-invasive ventilation, Long-term outcome

## Abstract

**Background:**

Non-invasive ventilation (NIV) is used to treat acute hypercapnic respiratory failure (AHRF) in patients with chronic obstructive pulmonary disease (COPD); however, long-term outcomes following discharge are largely unknown. This study aimed to characterize long-term outcomes and identify associated markers in patients with COPD after surviving the first episode of HRF requiring NIV.

**Methods:**

This study retrospectively analyzed 122 patients, mean age 62 ± 8 years, 52% female and forced expiratory volume in 1 s (FEV1) predicted 30 ± 13%, admitted with an acute hypercapnic exacerbation of COPD and receiving a first-ever NIV treatment between 2000 and 2012.

**Results:**

A total of 40% of the patients required hospital readmission due to respiratory reasons within 1 year. Persistent hypercapnia leading to the prescription of domiciliary NIV, older age and lower body mass index (BMI) were risk factors for readmission due to respiratory reasons. Survival rates were 79% and 63% at 1 and 2 years after discharge, respectively. A shorter time to readmission and recurrent hypercapnic failure, lower BMI and acidemia on the first admission, as well as hypercapnia at hospital discharge were correlated with a decreased long-term survival.

**Conclusion:**

Patients with COPD surviving their first episode of AHRF requiring NIV are at high risk for readmission and death. Severe respiratory acidosis, chronic respiratory failure and a lower BMI imply shorter long-term survival.

**Electronic supplementary material:**

The online version of this article (10.1007/s00508-018-1364-6) contains supplementary material, which is available to authorized users.

## Take home message

- Patients with COPD surviving their first episode of hypercapnic respiratory failure (AHRF) requiring non-invasive ventilation (NIV) are at high risk for readmission and death
- Severe respiratory acidosis, chronic respiratory failure and a lower BMI imply shorter long-term survival

## Introduction

Chronic obstructive pulmonary disease (COPD) is the third most common cause of death worldwide, resulting in an enormous burden on patients and on healthcare systems [1]. Mortality is increased in both the short-term and long-term period following an acute exacerbation of the disease [2]. Patients with COPD and acute respiratory failure face a high in-hospital mortality of 17–49% [3–5, 8]. Exacerbations become more frequent and more severe as COPD progresses [6]. The course of COPD involves a rapid decline in health status after the second severe exacerbation and high mortality in the weeks following a severe exacerbation [7]. Long-term mortality rate during the 4 years following hospital admission for acute exacerbation of COPD is high (45%) [9]. Severe exacerbations with acute hypercapnic respiratory failure (AHRF) may warrant admission to the intensive care unit (ICU) for non-invasive ventilation (NIV) [10]. Patients surviving such a respiratory crisis are at high risk for readmission and death [5, 11]. Associated markers of outcome following hospitalization for exacerbations of COPD were reviewed extensively by Steer et al. in 2010 [12] and Singanayagam et al. in 2013 [2]. In patients requiring mechanical ventilation, the available literature focused almost exclusively on long-term outcome after COPD exacerbations treated with invasive ventilation in patient cohorts derived from the 1990s. Either no or inconsistent potentially predictive markers for long-term outcomes were identified in these studies [13–22]. Few studies focused on the outcome following primary NIV, the current therapeutic gold standard. In particular, the long-term outcome in COPD patients surviving a hypercapnic exacerbation treated with NIV on the intensive care unit (ICU) has not been given sufficient attention to date. Therefore, the aim of this study was to characterize the long-term outcome after hypercapnic COPD exacerbations requiring NIV in the ICU and to investigate associated markers.

## Methods

### Setting

The Department for Respiratory and Critical Care at the Otto Wagner Hospital is a pulmonary tertiary care center in Vienna, Austria, providing a broad range of pulmonary care including an intensive care unit (ICU). The ICU receives patients from emergency services, affiliated hospitals and from the wards within the Otto Wagner Hospital. The use of NIV for acute hypercapnic exacerbations of COPD is provided only in the ICU and not on regular wards at this hospital. Therefore, all patients acutely requiring NIV are admitted to the ICU. For those patients receiving a prescription of domiciliary NIV following discharge, training in the use of home NIV is provided on our respiratory monitoring unit (RMU).

### Patients

The ICU records where searched for patients admitted with a diagnosis of COPD. Patients admitted to the ICU for NIV were included if the following criteria were met: age >18 years at index admission, underlying diagnosis of COPD as judged by the admitting physician, acute hypercapnic respiratory failure (AHRF; pH < 7.35, paCO2 > 45 mm Hg), primary treatment with non-invasive ventilation (with or without secondary endotracheal intubation) and survival to hospital discharge. All patients received standard medical care for COPD exacerbations based on the Global Initiative for Chronic Obstructive Lung Disease (GOLD) clinical guidelines.

Patients were excluded in the presence of the following: cardiopulmonary resuscitation or surgery immediately prior to the admission to the ICU, respiratory failure due to other causes (e. g. pulmonary embolism, acute myocardial infarction, asthma, bronchiectasis, bilateral pneumonia), prior resection of more than one lung lobe, obstructive sleep apnoea (OSAS), obesity hypoventilation syndrome (OHS), active cancer and congestive heart failure (CHF), i. e. history of hospitalization due to CHF, echocardiographic evidence of reduced left ventricular ejection fraction.

### Ethics

The study was conducted according to the principles of the Declaration of Helsinki. The ethics committee of the city of Vienna granted an exemption from requiring ethics approval (reference number EK 09-197_NZ). Given the retrospective, observational design of the trial, the need for informed consent was waived.

### Study design

The main study objective was to identify prognostic markers for long-term outcome in COPD patients surviving their first episode of HRF requiring NIV. Prognostic markers for predefined endpoints including survival (time between discharge and death), readmission due to respiratory reasons and recurrence of AHRF necessitating readmission to the ICU with a further episode of ventilatory support were sought. The following data sets were collected from the index admission, at hospital discharge and during follow-up:

#### Data from index admission

- Age
- Sex (male/female)
- Height (cm), body weight (kg), body mass index (BMI)
- History of previous intubation (y/n)
- Date of ICU admission
- COPD stage as based on lung function during a stable period within ±3 months of admission (FEV1/FVC ratio <70% and FEV1%, whereby Forced Vital Capacity [FVC])
- Simplified Acute Physiology Score (SAPS) II + estimated mortality
- Secondary Intubation (y/n), date of intubation, days spent intubated
- Days spent on non-invasive ventilation (NIV)
- Arterial blood gas (ABG) on admission including pH, CO2, FiO2, paO2 and paO2/FiO2 ratio
- Respiratory rate at the institution of NIV
- Highest C‑reactive protein (CRP) value within 3 days of admission (mg/dl)
- Use of hemodiafiltration (y/n)
- Maximum Therapeutic Intervention Scoring System (TISS28) score
- Presence of ICU-acquired pneumonia (y/n)
- Haemoglobin on admission (g/dl)
- Inspiratory Positive Airways Pressure (IPAP) and Positive End-Expiratory Pressure (PEEP) settings used at initiation of NIV
- Date of ICU discharge, length of ICU stay.

#### Data on hospital discharge

- Date of hospital discharge, length of hospital stay
- Arterial blood gas (ABG) at discharge
- Prescription of Long Term Oxygen Therapy (LTOT) ± NIV on discharge

#### Endpoints during follow-up

- Hospital readmission due to respiratory causes (y/n, date), whereas the term “respiratory reasons” was used as a header for any readmission due to an underlying pathology in the respiratory system (e. g. pneumonia, bronchitis, exacerbation of COPD, pulmonary embolism), also including recurrent acute hypercapnic respiratory failure (AHRF)
- Death (y/n, date)
- Last date seen alive

#### Definitions

- Acute hypercapnic respiratory failure (AHRF) = respiratory acidosis*:* pH < 7.35, paCO2 > 45 mm Hg
- Acidemia: pH < 7.35
- Hypoxemia: paO2/FiO2-ratio < 300
- Hypercapnia = hypercapnic respiratory failure: paCO2 > 45 mm Hg
- Persistent hypercapnia: paCO2 > 45 mm Hg at discharge

The study was conducted as an investigator-initiated retrospective record review. The index admission was confined to a single center. Follow-up data were derived from a total of 11 centers within the Vienna Hospital Association (Wiener Krankenanstaltenverbund, KAV), whereby there was no routine outpatient follow-up scheduled for the patients. All eligible patients treated within the study period were included. Survival time was defined by a patient’s vital status. All patients not reported as deceased during the follow-up were considered to be alive.

### Data collection

Data were extracted from electronic health records including the local ICU systems (medis, ICUdoc) as well as the city-wide health database (web.okra) of the Vienna Hospital Association. Data were anonymized and entered into an access database by three independent investigators. All the available data in the ICU health records going back to 2000 were used. Data collection was drawn to a close in 2012 when it was felt that enough patients were included to proceed to statistical analysis. It was chosen not to go back further than 2000 because of a shift in treatment paradigms (patients were more readily intubated in the 1990s). The city-wide health database was only used for follow-up.

### Analysis

Data are presented as median and interquartile ranges or as mean and standard deviation (±SD). After completion of data collection, data were cleaned and outliers (>95% confidence interval) were reconfirmed or corrected. Data were exported to a statistical software package (SPSS for Windows, version 15; SPSS Inc; Chicago, IL, USA) for analysis. Univariable data were described using standard methods. Association between risk factors and endpoints were assessed using bivariable and multivariable logistic regression (for dichotomous outcomes) or Cox regression (for time-dependent outcomes). In order to address collinearity, the automatic variable selection algorithm of SPSS was used: all potential associated markers (see methods section) were entered in the model but only those variables kept by both the forward and the backward conditional selection algorithm were included in the final models. Kaplan-Meier curves were used to demonstrate survival and readmission characteristics. Statistical significance was defined as *p* < 0.05.

## Results

There were 939 ICU admissions with a recorded diagnosis of COPD during the study period between February 2000 and June 2012. A COPD was regularly found as a comorbidity and not as the main reason for admission, which lead to the exclusion of the majority of cases (817 out of 939 admissions). Reasons for exclusion and respective frequencies are shown in Table 1. Only the index admission of an individual patient was considered for inclusion and repeat admissions were attributed to follow-up data.

**Table 1:** Reasons for excluded admissions

Exclusion criteria	Number of admissions (% of all excluded admission)
Surgery within 48 h prior to ICU admission	188 (23)
Respiratory failure due to other causes	146 (18)
AHRF criteria not met	88 (11)
Congestive heart failure	81 (10)
Primary intubation	78 (10)
OSAS	66 (8)
Other/insufficient data	52 (6)
Death before hospital discharge	45 (6)
Active cancer	45 (6)
CPR within 7 days prior to ICU admission	28 (3)

*AHRF* acute hypercapnic respiratory failure, *OSAS* obstructive sleep apnoea syndrome, *CPR* cardiopulmonary resuscitation, *ICU* intensive care unit

A total of 122 individual patients were included. Sex was evenly distributed, mean age was 62±8 years and mean BMI was 25±6. Spirometry was available in about half of the patients and indicated an emphasis on severe and very severe COPD stages with a mild degree of chronic hypercapnia. Of the patients 7 (6%) had a record of previous intubation due to e. g. pneumonia or sepsis, whereas none of these 7 patients had a record of previous intubation due to an exacerbation of COPD at this institution. Of the patients 5 (4.1%) were on domiciliary NIV prior to the admission. Demographics and spirometry results are outlined in Table 2.

**Table 2:** Demographics and spirometry results

Demographics	Patients	*n* = 122
	Sex	Female: *n* = 63 (52%)
	Age	62 (±8) years
	Height	166 (± 9) cm
	Weight	69 (±19) kg
	Body mass index	25 (±6)
	History of previous intubation	*n* = 7 (6%)
	Prior use of home NIV	*n* = 5 (4%)
Spirometry within ±3 months of index admission^a^	COPD stage	I: *n* = 0 II: *n* = 5 (7%) III: *n* = 19 (26%) IV: *n* = 48 (67%)
	FEV1 percent predicted	30 (±13) %
	Vital capacity	58 (±18) %
	Total lung capacity	139 (±26) %
ABG at time of spirometry^a^	pH	7.4 (±0.4)
	paO2	63 (±12) mm Hg
	paCO2	49 (±10) mm Hg

*FEV1* forced expiratory volume in 1 s, *ABG* arterial blood gas, *COPD* chronic obstructive pulmonary disease, *NIV* non-invasive ventilation

^a^spirometry within 3 months of index admission was available in 46.7% of the patients

Arterial blood gas analyses on admission showed acidemia, hypercapnia and hypoxemia (pH 7.25 ± 0.07; paCO_2_ 77 ± 17 mm Hg; paO_2_/FiO_2_ ratio 256 ± 115; Table 3). Radiographic evidence of a unilateral pneumonic infiltrate was present in 10% of the patients. All patients were started on NIV using an assisted mode with moderate pressure settings (IPAP 19 ± 4 mbar, PEEP 6 ± 2 mbar). A respiratory rate of 22 (±8) per minute was recorded at the initiation of NIV and following initial pharmacological management. The NIV failed in 18 patients (15%), necessitating secondary endotracheal intubation. No patient developed ventilator-associated or hospital-acquired pneumonia, sepsis or multi-organ failure.

**Table 3:** Data from the ICU admission

Arterial blood gases upon admission	pH	7.25 (±0.07)
	paO2	112 (±65) mm Hg
	paCO2	77 (±17) mm Hg
	FiO2	0.45 (±0.15)
	paO2/FiO2 ratio	256 (±115)
Respiration/ventilation	Respiratory rate	22 (±8)
	IPAP—inspiratory positive airway pressure	19 (±4) mbar
	PEEP—positive end-expiratory pressure	6 (±2) mbar
	NIV duration	5 (2–10) days
	Secondary intubation	18 patients (15%)
	Days spent intubated	5 (2–7) days
Laboratory results	Hemoglobin	13.5 (±2) g/dl
	CRP	38 (10–95) mg/l
	Lactate	2.7 (±1.5) mmol/l
ICU Scores	SAPS II score	34 (±11)
	SAPS II predicted mortality	14 (8–22) %
	TISS 28	30 (±6)

*ABG* arterial blood gas, *FiO2* fraction of inspired oxygen, *paO2/FiO2 ratio* ratio of paO2 over FiO2, *CRP* C-reactive protein, *NIV* non-invasive ventilation, *SAPS II* Simplified Acute Physiology Score II, *TISS 28* Therapeutic Intervention Scoring System

**Fig. 1:**
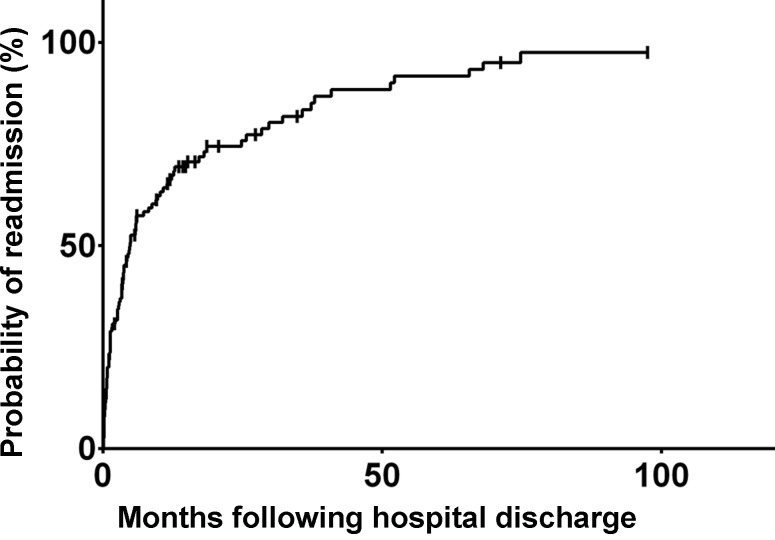
Probability of readmission due to respiratory reasons following discharge at index admission

**Fig. 2:**
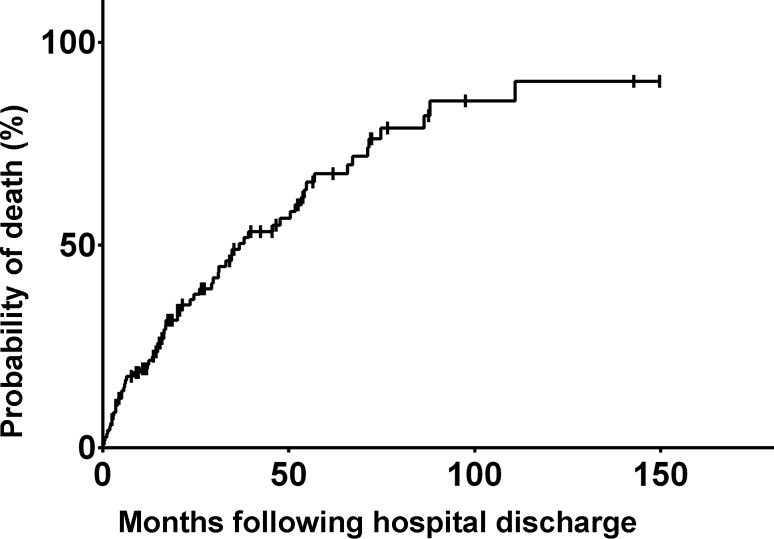
Probability of death following discharge at index admission

The median hospital and ICU length of stay was 19 days (range 14–27 days) and 7 days (3–11 days), respectively. Arterial blood gas readings at hospital discharge were pH 7.4 (±0.05), paO_2_ 81 (±23) mm Hg and paCO_2_ 53 (±11) mm Hg. Of the patients 85 (70%) received long-term oxygen treatment on hospital discharge and 26 patients (21%) were prescribed domiciliary NIV. Follow-up duration was defined as time from the index admission to the last documented patient contact (i. e. hospital readmission, out-patient follow up or death). Median follow-up duration was 1117 days (663–1570 days). Overall readmission rates due to any respiratory reason including recurrent hypercapnic respiratory failure were 40% (29–51%) at 1 year, 46% (35–59%) at 2 years and 73% (58–87%) at 5 years. Median time to readmission due to respiratory reasons was 112 days (33–328 days; Fig. 1). Survival rates were 79% (72–86%) at 1 year, 63% (53–73%) at 2 years and 32% (21–43%) at 5 years (Fig. 2). Median time of survival was 536 days (24–1337 days). The city-wide health database of Vienna includes all deaths, therefore there is no loss to follow-up with respect to death. Of the patients three (2.5%) were lost to follow-up with respect to the endpoint readmission and/or HRF.

### Associated markers of readmission and death

#### Associated markers of readmission to due respiratory reasons with or without hypercapnic respiratory failure

Multivariable Cox regression showed the following risk factors to be independently associated with a lower survival: lower BMI, higher age and lower paO2/FiO2 ratio on admission and a higher paCO2 on hospital discharge (Table 4).

**Table 4:** Associated markers of readmission to due respiratory reasons with or without hypercapnic respiratory failure in multivariable Cox regression

	Hazard ratio	*p* value
Lower BMI	0.96 (0.92–0.99)	0.019
Higher age, years	1.02 (1.00–1.05)	0.060
Lower paO2/FiO2-ratio	0.997 (0.996–0.999)	0.009
Persistent hypercapnia leading to prescription of home NIV	1.97 (1.21–3.22)	0.006

*BMI* body mass index, *NIV* non-invasive ventilation

#### Associated markers of overall survival

Multivariable Cox regression showed the following risk factors to be independently associated with a lower survival: lower BMI, lower pH and lower paO2/FiO2-ratio on admission and a higher paCO2 on hospital discharge (Table 5).

**Table 5:** Associated markers of overall survival in multivariable Cox regression

	Hazard ratio	*p* value
Lower BMI	0.94 (0.91–0.98)	0.0055
Lower pH	0.003 (0.000–0.187)	0.0054
Lower paO2/FiO2 ratio	0.997 (0.995–1.000)	0.0183
Higher paCO2 on discharge	1.030 (1.005–1.056)	0.0204

*BMI* body mass index

## Discussion

This article presents the first European study with an exclusive focus on long-term outcomes and associated markers in COPD patients surviving an episode of HRF requiring NIV in the ICU. This patient cohort faces a substantial long-term mortality with high rates of hospital readmission and recurrent hypercapnic respiratory failure. Of the patients 40% were readmitted due to respiratory reasons within 1 year. Survival rates were 79% and 63% at 1 and 2 years after discharge, respectively. Cox regression showed the use of domiciliary NIV, age, cachexia and hypoxemia on admission to be correlated with a higher likelihood of readmission due to respiratory reasons. A shorter time to readmission and recurrent hypercapnic failure, cachexia, acidemia, hypoxemia on admission, as well as hypercapnia at discharge were correlated with an increased long-term mortality.

A range of markers as outlined above were found to be associated with long-term outcomes. It is remarkable to find an association between markers of acute physiology and long-term outcomes as shown in the association of the level of acidosis and hypoxemia on admission and lower long-term survival. It is speculated that a more severe derangement of acute physiology points towards exhausted compensatory mechanisms, which might indirectly influence long-term outcome. A range of candidate markers did not show a correlation with long-term markers in this cohort. Most notably the need for secondary intubation, COPD stage, SAPS3 II and TISS 28 scores, days spent on ventilation and length of hospital stay did not show a correlation with long-term outcomes. In this respect it should be emphasized that the sample exclusively comprised survivors of the acute hospitalization episode whereas patients not surviving to hospital discharge were excluded.

Few studies are available in the literature for direct comparison to this study. In a large Canadian study on long-term outcome following the first hospitalization for a COPD exacerbation, mortality rates were 50% at 3.6 years and 75% at 7.7 years [7]. Unsurprisingly, mortality following COPD exacerbation requiring NIV, as reported in this study, is even higher. There is a bulk of studies on in-hospital and long-term outcomes following invasive ventilation in COPD patients, derived from study periods mostly stemming from the 1990s; however there has been a major shift in care towards primary NIV [10] and the prevalence and prognosis of COPD in critically ill patients has changed substantially [23]. The use of NIV has increased significantly over time among patients hospitalized for acute exacerbations of COPD, whereas the need for intubation and in-hospital mortality has declined [24].

A total of three papers were identified with a research question comparable to this study [11, 25, 26]. The sample size ranged between 93 and 110 patients in these studies. Chu et al. [11] published data from an Asian cohort receiving NIV in a respiratory care unit (RCU) and 1 year after discharge 79.9% had been readmitted, 63.3% had another life-threatening event and 49.1% had died. There are notable differences in the patient characteristics in comparison to the present study. For example, the Chu et al. cohort consisted predominantly of men (87 male and 23 female patients), while sex was evenly balanced in our patients. The subjects in the Chu et al. study were on average a decade older (mean age 73.2 vs. 62 years), more likely to be cachectic (mean BMI 20 vs. 25 in this study) and half of the patients had evidence of a pneumonic infiltrate compared to 10% in this study. Furthermore, Chu et al. included patients with prior episodes of intra-hospital NIV use whereas this study focused solely on first time application of NIV. There was no prescription of domiciliary NIV following discharge in the Chu et al. cohort while one in five of patients in this study was prescribed domiciliary NIV because of persistent hypercapnia. Given these differences in patient characteristics, only limited conclusions can be drawn from the direct comparison to the study of Chu et al. Echave-Sustaeta et al. [26] reported a study from Madrid including a cohort of 93 COPD patients (mean age 70.7 years, 83% male) surviving an episode of hypercapnia with ward-based NIV. Of the patients 66% required readmission within the following year and 1‑year survival was 69%. Chung et al. [25] studied 100 patients in an Australian cohort (mean age 70.6 years, 56% male) also receiving ward-based NIV where 56% needed readmission within 1 year and survival rates at 2 and 5 years were 52% and 26%, respectively. Potentially predictive markers that were identified in these studies are listed in table 6 in the online supplementary material. There is little inter-study reproducibility of potentially predictive markers. The only markers that were reproduced in another study were arterial pH prior to initiation of NIV and age in association with long-term mortality. Long-term survival is consistently poor across the 4 studies, with only 1 in 4 patients surviving 5 years following discharge. Given the high morbidity and mortality in COPD patients surviving an episode of HRF, it seems mandatory to discuss the prognosis with the patients and to elicit their wishes concerning readmission to ICU and possible reinstitution of ventilatory support.

The present study is limited by its retrospective design and a relatively small sample size. Furthermore, data on smoking status could not be obtained, LTOT prior to admission, length of stay in hospital in the year prior to admission, the Medical Research Council (MRC) dyspnea score and the Katz Index of Independence in Activities of Daily Living score (some of these factors have been identified as associated markers in other studies, as outlined in the online supplementary material). Spirometry within 3 months of admission was available in only half of the patients. Readmissions outside Vienna or in a private hospital within Vienna might have been missed. In an attempt to define this patient cohort as precisely as possible, relatively rigid exclusion criteria were used. It is acknowledged that this might have introduced some bias due to patient selection. The COPD is regularly found with a range of comorbidities that might themselves lead to respiratory failure. It was decided to attribute the cause of respiratory failure not solely to COPD (and therefore to exclude the patient) when there was recorded evidence of an acute illness that by itself may be reason enough to cause respiratory failure. It is acknowledged that it is difficult to adequately draw the line here in the presence of several comorbidities. As stated, all patients requiring NIV are admitted to the ICU at this hospital. The results may not be transferable to hospitals where NIV is also provided on regular wards.

The data point towards a lower BMI and chronic hypercapnia as targets for clinical intervention. Weight gain improves survival in undernourished patients with COPD [27]; however, methods to achieve a reliable weight gain in COPD patients remain elusive. The addition of long-term NIV to standard treatment improves survival of patients with stable hypercapnic COPD when NIV is targeted to greatly reduce hypercapnia [28]. In seems likely that these findings from stable COPD patients would also be observed in patients following acute hypercapnic respiratory failure. Struik et al. randomized 201 COPD patients with prolonged hypercapnia after ventilatory support for acute respiratory failure to either nocturnal NIV or standard care. An improvement in daytime pCO2 and trend to a better quality of life was found but no effect on mortality or readmission rate was seen after 1 year [29]. In patients who used NIV following an admission for an acute exacerbation of COPD (AECOPD) with AHRF, Galli et al. on the other hand found lower readmission rates and an improved event-free survival after 180 days from an index admission compared to patients who did not use NIV post-discharge [30].

In conclusion, there is substantial morbidity and mortality in this patient population. Promising targets for intervention identified in this study, in line with previously published studies, are a low BMI and chronic hypercapnia. The authors believe that these findings should be validated in a prospective cohort leading to predictive models that can be used to facilitate the shared decision-making process.

## Conclusion

Patients with COPD surviving the first episode of HRF requiring NIV are at high risk for readmission and death. Distinct risk factors associated with long-term outcomes were identified; however, there is substantial inconsistency among studies published in this field. Results from this study point towards a low BMI and chronic hypercapnia as possible targets for therapeutic intervention.

## Caption Electronic Supplementary Material

Table 6 Comparison of study characteristics and identified prognostic markers in other studies
