# Supplementary material for: Long-term outcomes after acute hypercapnic COPD exacerbation: First-ever episode of non-invasive ventilation
Source: Wien Klin Wochenschr. 2018 Jul 31;130(19):561–8. doi: 10.1007/s00508-018-1364-6 (PMC6209011; doi:10.1007/s00508-018-1364-6)
Supplement: Supplementary file 1 — Table 6 Comparison of study characteristics and identified prognostic markers in other studies [file 508_2018_1364_MOESM1_ESM.doc]

**ONLINE SUPPLEMENTARY MATERIAL**

| **Outcome** | **Chu 2004 (n=110, RCU)** | **Echave-Sustaeta 2010 (n=93, ward-based)** | **Chung 2010**  **(n=100, ward-based)** | **Fazekas 2018**  **(n=122, ICU)** |
| --- | --- | --- | --- | --- |
| **Readmission due to respiratory reasons** | - days in hospital in the previous year (U,M)  - Katz score (U,M) - DNI status (U) | - paCO2 prior to initiation of NIV (U)  - FEV1% (U,M)  - length of stay during hospitalisation (U,M) | - male sex  - home oxygen | - home NIV following discharge (U,M)  - age (M)  - BMI (M)  - paO2/FiO2-ratio (M) |
| **Mortality** | - MRC dyspnoea score (M) | - age (U,M) - pH prior to NIV (U) paCO2 prior to NIV (U, M) - days in hospital in the previous year (M) - home NIV after discharge (U) - length of hospital stay (U) | - age (U,M)  - prior LTOT (U,M)  - BMI (U,M) | - time to readmission due to respiratory reasons (U)  - time to recurrent AHRF (U)  - BMI (M)  - pH (M)  - paO2/FiO2-ratio (M)  - paCO2 on discharge (M) |

*Table 6 Comparison of study characteristics and identified prognostic markers in other studies DNI – do not intubate*, *MCR – Medical Research Council*, *APACHE – Acute Physiology and Chronic Health Evaluation*, *LTOT – Long-Term Oxygen Therapy*, *U – Predictor identified in univariable analysis*, *M - Predictor identified in multivariable analysis*
